# Expression of the eight GABA_A_ receptor α subunits in the developing zebrafish central nervous system

**DOI:** 10.1371/journal.pone.0196083

**Published:** 2018-04-27

**Authors:** Bryan Monesson-Olson, Jon J. McClain, Abigail E. Case, Hanna E. Dorman, Daniel R. Turkewitz, Aaron B. Steiner, Gerald B. Downes

**Affiliations:** 1 Neuroscience and Behavior Graduate Program, University of Massachusetts, Amherst, MA, United States of America; 2 Biology Department, University of Massachusetts, Amherst, MA, United States of America; 3 Department of Biology and Health Sciences, Pace University, Pleasantville, NY, United States of America; National Institutes of Health, UNITED STATES

## Abstract

GABA is a robust regulator of both developing and mature neural networks. It exerts many of its effects through GABA_A_ receptors, which are heteropentamers assembled from a large array of subunits encoded by distinct genes. In mammals, there are 19 different GABA_A_ subunit types, which are divided into the α, β, γ, δ, ε, π, θ and ρ subfamilies. The immense diversity of GABA_A_ receptors is not fully understood. However, it is known that specific isoforms, with their distinct biophysical properties and expression profiles, tune responses to GABA. Although larval zebrafish are well-established as a model system for neural circuit analysis, little is known about GABA_A_ receptors diversity and expression in this system. Here, using database analysis, we show that the zebrafish genome contains at least 23 subunits. All but the mammalian θ and ε subunits have at least one zebrafish ortholog, while five mammalian GABA_A_ receptor subunits have two zebrafish orthologs. Zebrafish contain one subunit, β4, which does not have a clear mammalian ortholog. Similar to mammalian GABA_A_ receptors, the zebrafish α subfamily is the largest and most diverse of the subfamilies. In zebrafish there are eight α subunits, and RNA *in situ* hybridization across early zebrafish development revealed that they demonstrate distinct patterns of expression in the brain, spinal cord, and retina. Some subunits were very broadly distributed, whereas others were restricted to small populations of cells. Subunit-specific expression patterns in zebrafish resembled were those found in frogs and rodents, which suggests that the roles of different GABA_A_ receptor isoforms are largely conserved among vertebrates. This study provides a platform to examine isoform specific roles of GABA_A_ receptors within zebrafish neural circuits and it highlights the potential of this system to better understand the remarkable heterogeneity of GABA_A_ receptors.

## Introduction

Neural networks throughout the central nervous system rely upon a diversity of neurotransmitter systems for both their initial formation and mature function. GABA is the major inhibitory neurotransmitter throughout most of the mature nervous system [1]. It exerts its effects through its receptors, which are divided into two classes, GABA_A_ and GABA_B_. GABA_A_ receptors are ligand-gated chloride channels responsible for most of the rapid effects of GABA, while GABA_B_ receptors are heterotrimeric G-protein coupled receptors. In mammalian systems, GABA_A_ receptors demonstrate incredible subtype diversity and are targets for classes of clinically important drugs, such as benzodiazepines and barbituates [2, 3]. GABA_A_ receptors are heteropentamers composed of various combinations of 19 different subunits: α1–6, β1–3, γ1–3, δ, ε, π, θ and ρ1–3 (previously referred to as GABA_c_ receptors) [4, 5]. Each subunit is encoded by a discrete gene that is spatially and developmentally regulated to generate distinct, but often overlapping, expression patterns [6–9]. Alternative splicing and RNA editing of some subunits further increases the number of subtypes available [10]. Although this extensive receptor heterogeneity is not fully understood, some subunits confer distinct biophysical and pharmacological properties, interact with specific cytoplasmic proteins, and localize to specific subcellular domains [11–13]. Ultimately, this receptor diversity provides a capacity to tailor responses to GABA within neural circuits.

The α subunits play a prominent role in GABA_A_ receptor function. They are thought to be essential components of ‘classic’ GABA_A_ receptors, which exclude the pharmacologically distinct receptors composed from ρ subunits [3]. Exogenous expression studies and analysis of native receptors indicate that each receptor contains two α subunits which, along with β subunits, form the GABA binding site. α subunits can also dictate isoform-selective pharmacology. For example, receptors containing α1, α2, α3 and α5 subunits confer benzodiazepine-sensitivity, whereas receptors that contain α4 and α6 subunits do not bind to clinically used benzodiazepines [2]. In addition, α subunits regulate subcellular localization. GABA receptors that contain α1, α2, or α3 demonstrate a synaptic localization and mediate transient or phasic inhibition, while receptors that contain α4, α5 or α6 are principally extrasynaptic and mediate tonic inhibition [14, 15]. Through both transient and tonic inhibition, these receptors mediate the majority of inhibition in neural circuits throughout the vertebrate brain.

Developing zebrafish have several features that make them well-suited to study the formation and function of neural circuits [16, 17]. First, zebrafish embryos and larvae develop external to the mother and are optically transparent. These features make the central nervous system easily accessible throughout development and particularly amenable to optical physiological approaches, such as optogenetics or calcium imaging. Second, many cell types and mechanisms are conserved among vertebrates, yet the central nervous system contains fewer cells compared to mammalian preparations. Lastly, larval zebrafish develop rapidly, therefore many sensory and motor systems are present and functional within five days post-fertilization. Combined, these features have helped establish developing zebrafish as an important system to examine neural circuit formation and function.

GABA_A_ receptors are expressed in larval zebrafish and essential for normal nervous system function. For example, bath application of pentylenetetrazole, a convulsant and noncompetitive GABA_A_ receptor antagonist, induces hyperactive behavior that can serve as a model of seizures [18]. Similarly, insecticides known to target GABA_A_ receptors generate hyperactive behavior in larvae [19]. Pharmacological blockade of GABA_A_ receptors has also been shown to regulate the activity of specific cells, including retinal bipolar cells [20], optic tectum neurons [21], mitral cells in the olfactory bulb [22], spinal cord CoPA interneurons and motoneurons [23], and the Mauthner cells, a pair of well-studied reticulospinal neurons found in the amphibian and teleost hindbrain [24]. In these studies, the GABA_A_ receptor isoforms were not identified. However, patch-clamp recordings of the Mauthner cells showed three distinct GABA_A_ kinetic profiles, which were proposed to be caused by different receptor isoforms [25].

There is limited information about the heterogeneity of GABA_A_ receptors in zebrafish. The most extensive study to date identified 23 GABA_A_ receptor subunits and examined their expression broadly in adult zebrafish brain via RT-qPCR [26]. Despite playing major roles in developing animals, little is known about the expression of GABA_A_ receptor subunits in zebrafish embryos and larvae. α1 has been shown to be expressed at 50 and 72 hours post-fertilization (hpf), γ2 at 50 hpf, and α2a (then named α2) from ~14–96 hpf. [27–29]. Unpublished α6a expression data has also been deposited in the ZFIN database [30]. The expression patterns of other GABA_A_ receptor subunits has not been reported.

In this study we performed phylogenetic analysis, which indicates that zebrafish contain at least 23 different GABA_A_ receptor subunits. Although we observed some differences between the zebrafish and mammalian GABA_A_ receptor subunit gene families, zebrafish contain orthologs for most of the GABA_A_ receptor subunits found in mammals. To examine when and where these GABA_A_ receptors are expressed in the zebrafish nervous system, we performed whole-mount RNA *in situ* hybridization. We present the embryonic and larval expression of the eight identified α subunit-encoding genes: *gabra1*, *gabra2a*, *gabra2b*, *gabra3*, *gabra4*, *gabra5*, *gabra6a* and *gabra6b*. These data show that each subunit has a distinct expression pattern, broadly similar to the reported expression of amphibian and rodent orthologs. Combined, these results suggest that GABA_A_ isoform-specific roles are conserved among vertebrates, and they establish a foundation to use the zebrafish system to better understand how GABA_A_ receptor heterogeneity mediates neural circuit formation and function.

## Materials and methods

### Phylogenetic analysis

Homologous gene queries were performed using the National Center for Biotechnology Information tBLASTn search tool. Protein sequences for each of the 19 mouse GABA_A_ receptor subunits were used to search the zebrafish nucleotide collection. Matching zebrafish sequences were evaluated to determine if they were splice variants from the same gene or generated from different genes. When splice variants were identified, only the longest variant was used for subsequent analysis. 19 mouse and 23 zebrafish protein sequences were used to assemble a phylogenetic tree. The genes and Genbank accession numbers for the mouse sequences are: *Gabra1*- NP_034380, *Gabra2*- NP_032092, *Gabra3*- NP_032093, *Gabra4*- NP_034381, *Gabra5*- NP_795916, *Gabra6*- NP_00109311, *Gabrb1*- NP_032095, *Gabrb2*- NP_032096, *Gabrb3*- NP_032097, *Gabrd*- NP_032098, *Gabre*- NP_059065, *Gabrg1*- NP_034382, Gabrg2- NP_032099, *Gabrg3*- NP_032100, *Gabrp*- NP_666129, *Gabrq*- NP_065234, *Gabrr1*- NP_032101, *Gabrr2*- NP_032102, and *Gabrr3*- NP_001074659. The Genbank accession numbers for the zebrafish sequences are: *gabra1*- NM_001077326, *gabra2a*- XM_009307207, gabra2b- XM_017359049, *gabra3*- XM_009295708, *gabra4*- NM_001017822, *gabra5*- XM_001339475, *gabra6a*- XM_005173112, *gabra6b*- XM_002667357, *gabrb1*- XM_002664133, *gabrb2*- NM_001024387, *gabrb3*- XM_005166079, *gabrb4*- XM_005173874, *gabrd*- XM_695007, *gabrg1*- XM009307208, *gabrg2*- NM_001256250, *gabrg3*- XM_009302568, *gabrp*- XM_005173293, *gabrr1*- NM_001025553, *gabrr2a*- NM_001045376, *gabrr2b*- XM_009294512, *gabrr3a*- NM_001128760, *gabrr3b*- XM_009297450, *gabrz*- XM_005156247. To construct the tree, a ClustalW alignment of amino acid sequences was performed using the Geneious software package [31]. An unrooted PHYML consensus tree was then generated using 100 bootstrap replicates [32].

### Fish maintenance and breeding

Zebrafish were raised and maintained using established husbandry procedures. Embryos were kept at 28.5 °C in E3 media and staged according to morphological criteria [33]. All experiments were performed using Tuebingen (Tu) or Tupfel long fin (TLF) wild type embryos. From 0–24 hpf, embryos were grown in 0.01% Methylene Blue in E3 medium. At 24 hpf the solution was changed to 0.0045% Phenylthiourea (PTU) in E3 to inhibit pigment formation. This solution was changed every 24 hours until the fish were sacrificed by anesthetizing them in 0.4% MS-222 until the cessation of movement then transferring the fish into 4% paraformaldehyde. All animal protocols were approved by the University of Massachusetts Institutional Animal Care and Use Committees (IACUC).

### Whole-mount in situ hybridization

Antisense digoxigenin probes were generated against *gabra1*, *gabra2a*, *gabra2b*, *gabra3*, *gabra4*, *gabra5*, *gabra6a* and *gabra6b* (S1 Table). *In situ* probes were synthesized using a digoxigenin RNA labeling kit with SP6 or T7 RNA polymerases (Roche Diagnostics, Mannheim, Germany). Whole-mount, colorimetric *in situ* hybridization was performed using established protocols [34] and examined using a compound microscope (Zeiss, Thornwood, NY) attached to a digital camera (Zeiss, Thornwood, NY). Cross sections were generated by imbedding *in situ* hybridization stained embryos in 1.5% agar, 5% sucrose. The blocks were submerged in a 30% sucrose solution overnight then cut into 20 μm thick sections using a cryostat (Leica, Buffalo Grove, IL). Representative larvae at 24, 48, and 96 hpf were mounted in either 100% glycerol or dehydrated through a methanol series, equilibrated in a 2:1 benzylbenzoate/benzylalcohol solution and mounted in a 10:1 Canada balsam/methyl salicylate mixture. Images were captured using a Zeiss Discovery v12 stereomicroscope with a 1.5x objective or a Zeiss Axioskop Microscope with a 10x objective and an AxioCam MRc camera. All images were processed using Adobe Photoshop, in which multiple focal planes were merged to produce single representative images. The identify of neuroanatomical structures was determined using zebrafish brain atlases [35–37].

### Reverse-Transcriptase PCR

RT-PCR was used to analyze whether the *gabra3* and *gabra3*-like sequence were portions of the same transcript. Primers designed against *gabra3*-like (Primer 1, 5’-GGACGGCGGATGATGAGAAA-3’) and *gabra3* (Primer 2, 5’-CACGACCGTCCTGACTA-3’, Primer 3, 5’-GTGGAGTAGATGTGGTGGGC-3’) were used to amplify cDNA from wild-type zebrafish larvae. RNA was extracted from 48 hpf zebrafish larvae using the RNAeasy kit (Qiagen, Venlo, Netherlands) and reverse transcribed using the Accuscript RT-PCR system (Stratagene). The PCR products were sequenced to confirm that they are portions from the same transcript.

## Results

### The zebrafish GABA_A_ receptor subunit gene family is similar in size and diversity to the mammalian GABA_A_ receptor gene family

**Fig 1:**
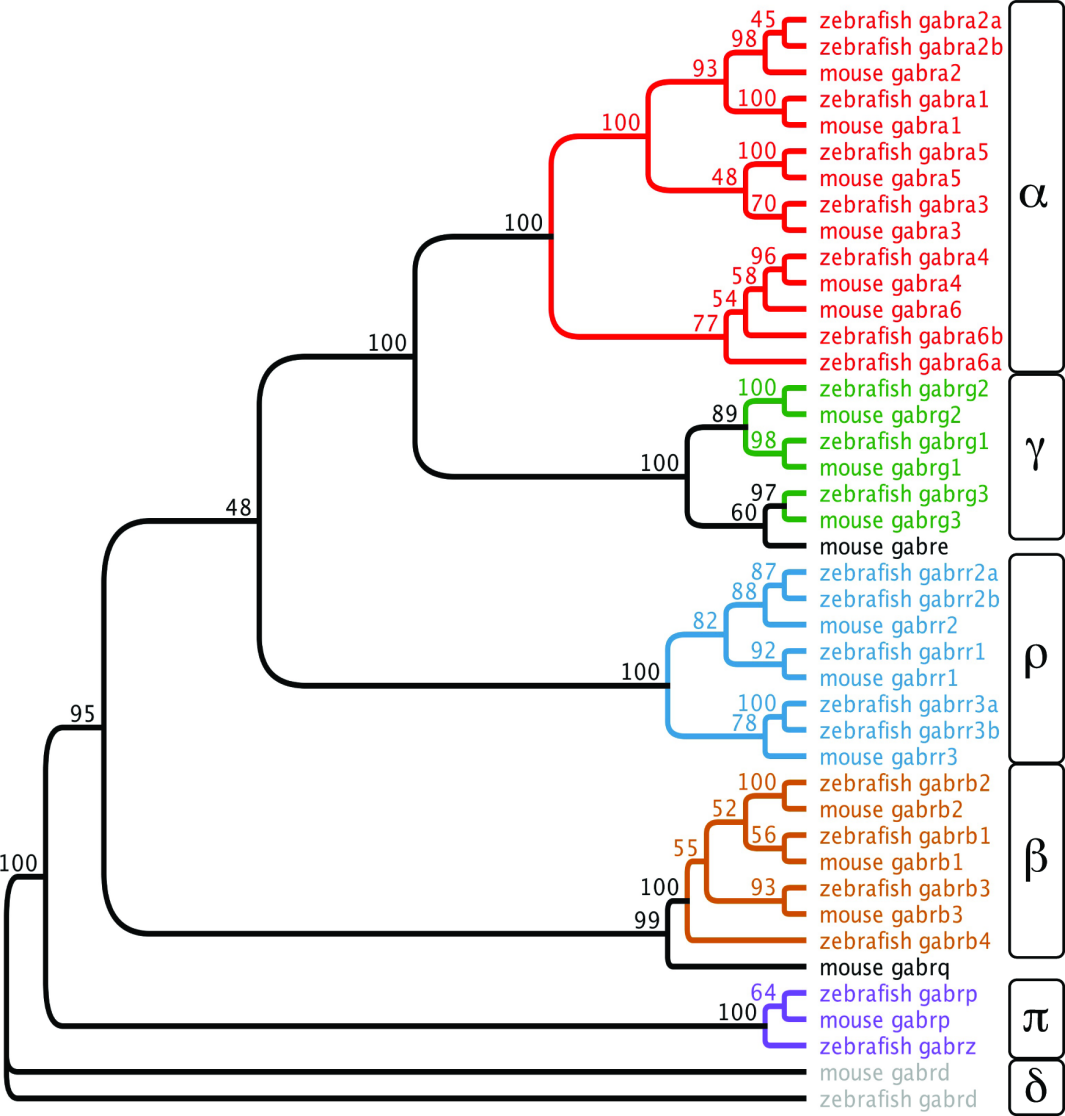
Phylogenetic analysis shows that the zebrafish GABA_A_ subunit gene family is similar in size, diversity, and organization to the mouse GABA_A_ subunit gene family. Amino acid sequence alignments were used to generate a consensus tree using 100 bootstrap replicates. The genes that encode the GABA_A_ subunits are shown at the tip of each branch and bootstrap proportions are shown at the branch points. The zebrafish and mouse GABA_A_ subunit sequences showed high amino acid identity and grouped into the α, β, γ, δ, π, and ρ subfamilies. Most mouse GABA_A_ subunits have a single zebrafish ortholog, while six mouse GABA_A_ subunits have two zebrafish orthologs, likely due to gene duplication.

19 GABA_A_ receptor subunit genes have been identified in humans, mice, and rats [4, 5]. To establish the number and organization of zebrafish GABA_A_ receptor subunits, we used mouse GABA_A_ receptor subunit amino acid sequences to query zebrafish genome databases. We identified 23 zebrafish GABA_A_ receptor subunits, each encoded by a distinct gene. Splice variants were frequently observed. In these cases, the principle splice isoform, as indicated by the databases, was selected for analysis. 12 of the 19 mouse GABA_A_ receptor subunits were found to have a single ortholog in zebrafish (Fig 1). Amino acid identities between mouse and zebrafish orthologs ranged from 53.1 to 86.3%. Five additional mouse subunits, α2, α6, ρ2, ρ3, and π, each exhibit similarity with two subunits in zebrafish: *gabra2a* and *gabra2b*, *gabra6a* and *gabra6b*, *gabrr2a* and *gabrr2b*, *gabrr3a* and *gabrr3b*, *gabrp* and *gabrz*, respectively. The relatively high percentage of amino acid identity of these zebrafish subunits with each other suggests that the zebrafish paralogs are duplicated genes. Duplicated genes are often observed in zebrafish, and they are thought to be due to a whole genome duplication within the teleost lineage [38, 39]. It was reported previously that there is a *gabra3* and an α3-like gene. While the α3-like gene has been localized to chromosome 21, the genomic location of the α3-like gene is not known. Our database analysis suggested that these sequences could be non-overlapping portions of the same gene, therefore RT-PCR was performed using primers targeting α3-like and *gabra3* sequences (S1 Fig). Transcripts were amplified that spanned the region between *gabra3* and α3-like sequences, indicating that these are different portions of the same gene and that there is only one α3 encoding gene, *gabra3*.

There are two mouse GABA_A_ receptor subunits with no clear orthologs, the θ and ε subunits. The θ subunit, encoded by the gene *Gabrq*, exhibits only 31.3% amino acid identity with zebrafish β4, encoded by *gabrb4*, which is the greatest percentage identity of this subunit with any zebrafish sequence. There are three β subunits in mice compared to the four β subunits in zebrafish so it is possible that, despite the low amino acid sequence identity, zebrafish β4 is orthologous to the mouse θ subunit. No clear zebrafish ortholog has been identified for the mouse ε subunit, which is encoded by the *Gabre* gene.

### α subunits demonstrate distinct expression patterns across early zebrafish development

Determining the temporal and spatial expression of GABA_A_ subunits is essential to identify isoform-specific roles in zebrafish neural circuit development and function. Given the large size of the α subunit subfamily and their leading role in GABA_A_ receptor function, we examined the expression of the eight α subunit-encoding genes: *gabra1*, *gabra2a*, *gabra2b*, *gabra3*, *gabra4*, *gabra6a* and *gabra6b*. Using whole-mount RNA *in situ* hybridization, we determined their expression patterns at 24, 48, and 96 hpf. These time points span key stages of locomotor network development in developing zebrafish [40].

**Fig 2:**
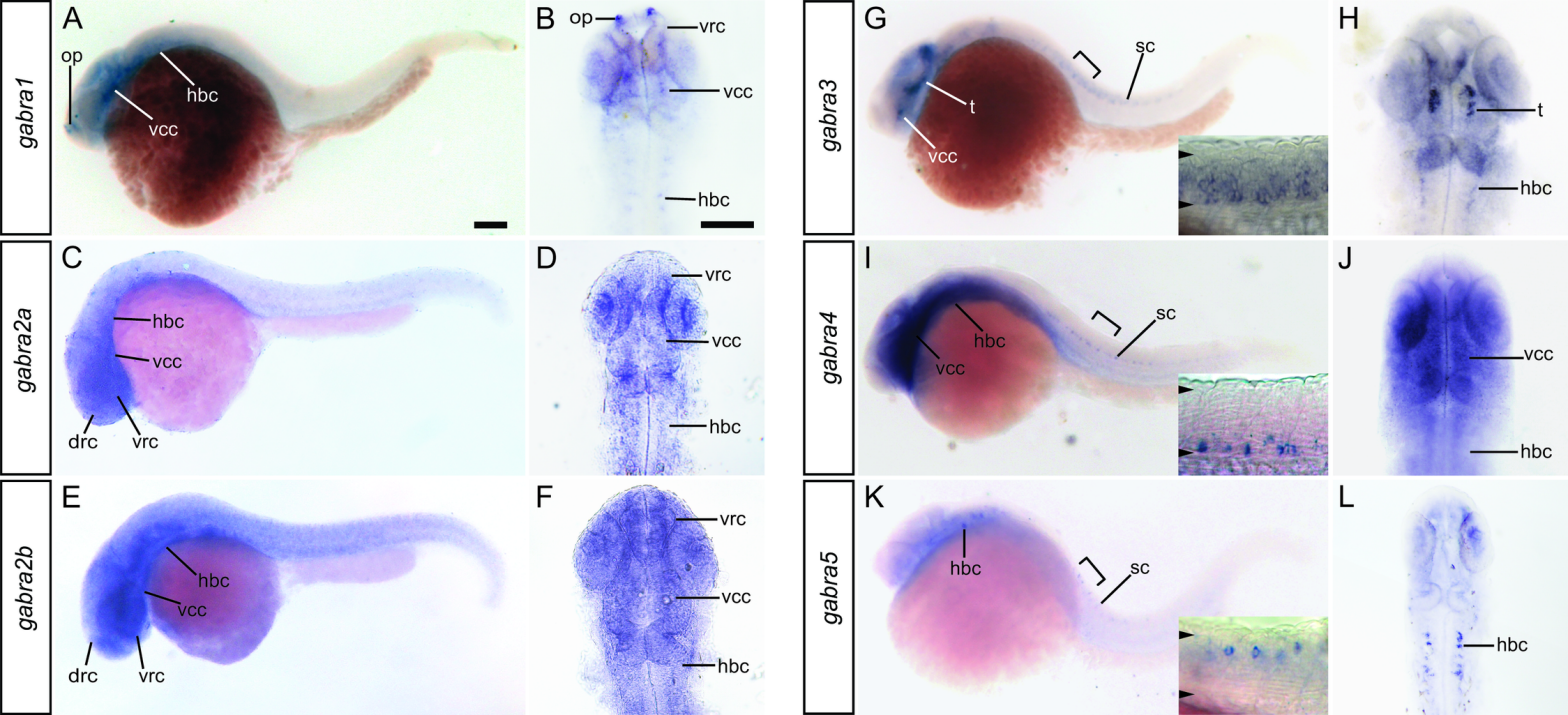
Expression of GABA_A_ α subunits at 24 hpf. *gabra1* (A, B), *gabra2a* (C, D), *gabra2b* (E, F), *gabra3* (G, H), *gabra4* (I, J), and *gabra5* (K, L) were detected at this time point. Whole-mount lateral (A, C, E, G, I, K) views are shown along with dorsal views of the head (B, D, F, H, J, L). The scale bar (A) is 0.1 mm. The brackets in G, I, and K indicate the regions shown at higher magnification in the corresponding insets. The *arrowheads* within the insets indicate the dorsal and ventral boundaries of the spinal cord. Abbreviations: drc, dorsal rostral cluster; hbc, hindbrain cluster; op, olfactory placode; SC, spinal cord; t, tegmentum; vcc, ventral caudal cluster; vrc, ventral rostral cluster.

Expression of *gabra1*, *gabra2a*, *gabra2b*, *gabra3*, *gabra4* and *gabra5* was detected at 24 hpf (Fig 2). Neither *gabra6a* or *gabra6b* were detected at this developmental stage. *gabra2a* and *gabra2b* were detected broadly and did not appear to be spatially restricted (Fig 2C–2F). To distinguish whether these gene transcripts were widespread or background due to the probe, a second probe was generated for each gene (S1 Table). These probes yielded widespread staining, very similar to the initial probes used, which suggests that *gabra2a* and *gabra2b* are widely distributed. Consistent with these findings, a previous study reported that *gabra2a* exhibits a diffuse, broad pattern of expression in embryonic and larval zebrafish [28].

*gabra1*, *gabra3*, *gabra4*, and *gabra5* were each expressed in discrete cells in the brain or spinal cord at 24 hpf. *gabra1* was detected in the olfactory placodes, the ventral rostral cluster, the ventral caudal cluster and small clusters of cells within each rhombomere of the hindbrain (Fig 2A and 2B). Although expressed in discrete cells, *gabra3* was widely expressed, and found within the tegmentum, hindbrain, and ventral and intermediate domains in the spinal cord (Fig 2G and 2H). *gabra4* was expressed in a diffuse manner throughout the brain but selectively within a population of ventral spinal cord cells (Fig 2I and 2J). These *gabra4* expressing cells are cuboidal in appearance and along the central canal, which suggests they may be Kolmer-Agduhr (KA) neurons [41]. Lastly, *gabra5* is expressed in small groups of cells in each rhombomere of the hindbrain and in a dorsal domain of the spinal cord (Fig 2K and 2L). The large soma size of these spinal cord cells, their dorsal position, and the number of labeled cells per somite (~1) suggests that they are CoPA neurons, which have been previously reported to receive GABAergic input [23, 42].

**Fig 3:**
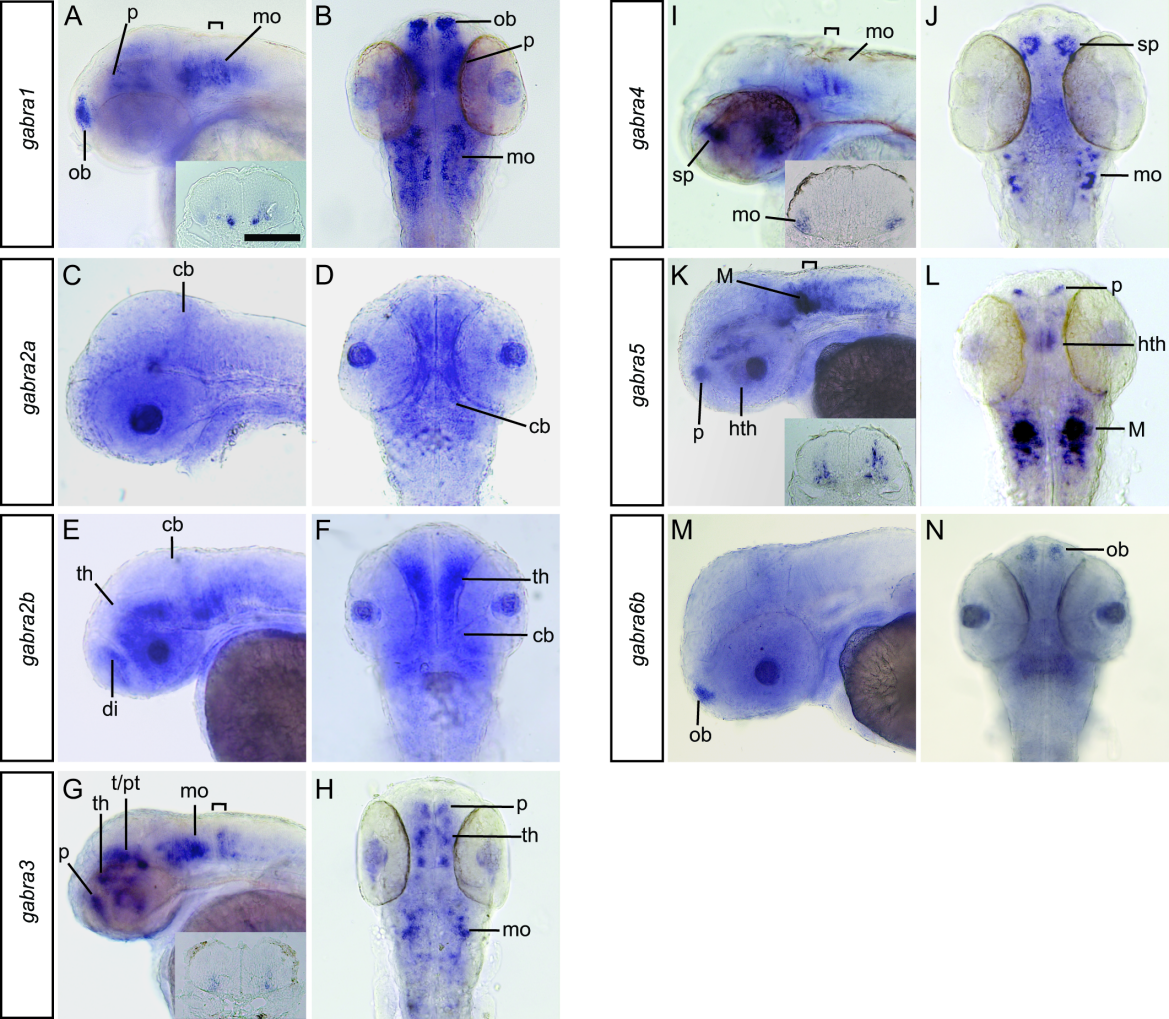
Expression of the GABA_A_ α subunits in the brain at 48hpf. *gabra1* (A, B), *gabra2a* (C, D), *gabra2b* (E, F) *gabra3* (G, H) *gabra4* (I, J) *gabra5* (K, L) and *gabra6b* (M, N) were detected at this time point. Lateral (A, C, E, G, I, K, M) and dorsal (B, D, F, H, J, L, N) views are shown. The scale bar (A) is 0.1 mm. Brackets in A, G, I, and K indicate the region shown in cross sections within the insets. Abbreviations: cb, cerebellum; di, diencephalon; hth, hypothalamus; M, Mauthner cell; mo, medulla oblongata; ob, olfactory bulb; p, pallium; sp, subpallium; t, tegmentum; th, thalamus.

The expression of all GABA_A_ receptor subunit genes, except for *gabra6a*, was observed at 48 hpf (Fig 3). Similar to the pattern observed at 24 hpf, the *gabra2* paralogs were again detected broadly, with little spatial restriction (Fig 3C–3F). Similar to its expression at 24hpf, *gabra1* is expressed in the olfactory bulbs, the pallium and discrete clusters of cells in medial portions of the medulla (Fig 3A and 3B). *gabra3* was observed in relatively small clusters of cells in the pallium, thalamus, and the medulla (Fig 3G and 3H). *gabra4* transcripts were identified in distinct cells in the subpallium and lateral potions of the medulla. *gabra5* transcripts were detected most prominently in the medulla. This staining suggests α5 is expressed in the Mauthner cells, and that α5 containing receptors are likely some of the GABA_A_ isoforms identified via electrophysiology [25]. *gabra6b* is expressed prominently in the olfactory bulbs.

Although *gabra1*, *gabra3*, *gabra4*, and *gabra5* transcripts were all detected in the medulla, they do not appear to be in the same cells. Instead, an intriguing medial to lateral organization was observed. *gabra1* is expressed strongly in the most medial cells, *gabra3* and *gabra5* transcripts were found in an intermediate domain, and *gabra4* was observed laterally (compare insets in Fig A, G, I, K).

**Fig 4:**
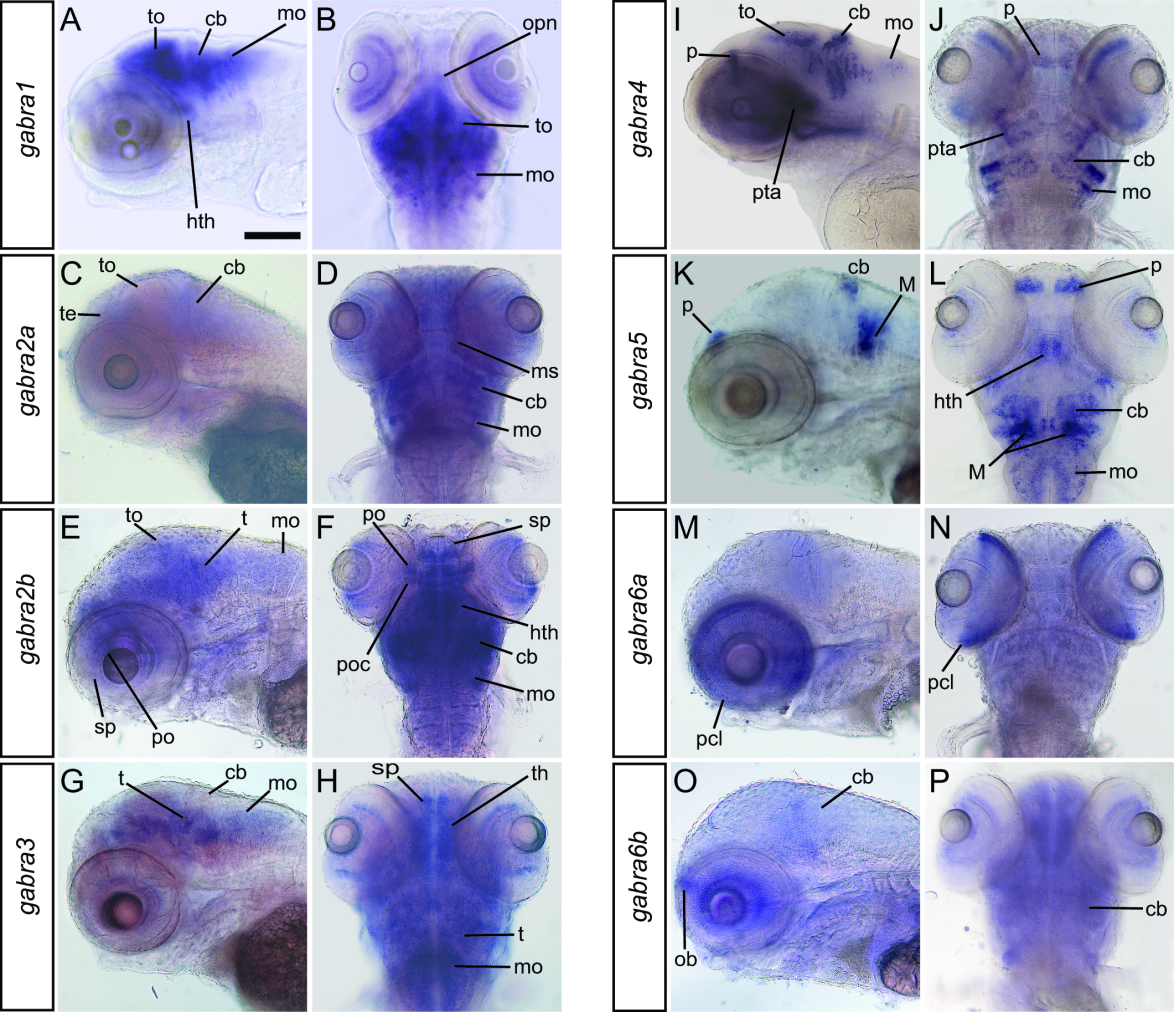
Expression of the GABA_A_ α subunits in the brain at 96hpf. Transcripts encoding all α subunits were detected at this time point. Lateral (A, C, E, G, I, K, M, O) and dorsal (B, D, F, H, J, L, N, P) are shown The scale bar (A) is 0.1 mm. Abbreviations: cb, cerebellum; gcl; hth, hypothalamus; M, Mauthner cell; mo, medulla oblongata; ob, olfactory bulb; opn, optic nerve; p, pallium; pcl, photoreceptive cell layer; po, preoptic region; poc, post optic commissure; pta, posterior tubercular area; sp, subpallium; t, tegmentum; te, telencephalon; th, thalamus; to, tectum opticum.

Transcripts for all α subunits are detected in larval zebrafish at 96 hpf (Fig 4). As with the earlier developmental time points, *gabra2a* and *gabra2b* transcripts were detected broadly, although *gabra2b* appears less diffuse and more discrete compared to at 24 and 48 hpf (Fig 4C–4F). *gabra1*, *gabra3*, and *gabra6b* are also expressed more broadly compared to earlier stages. In contrast, *gabra4*, *gabra5* and *gabra6a* transcripts were detected in smaller groups of cells. *gabra4* is expressed in the retina, the posterior tuberculum area, which is a portion of the diencephalon, and the tectum, cerebellum, and medulla (Fig I, J). *gabra5* is expressed in discrete cells in the pallium, hypothalamus, cerebellum and medulla (Fig K, L). Within the medulla, *gabra5* expression in the Mauthner Cells is robust, as it is at earlier developmental stages.

Transcripts for several α subunits are expressed in the retina at 96 hpf, however most are restricted to one or two cell layers (Fig 4). *gabra1*, *gabra2a*, *gabra2b*, *gabra3*, *gabra4*, and *gabra6b* transcripts are all expressed in distinct cell layers in the retina. Although most of these genes are also expressed in other areas of the nervous system, *gabra6a* does not demonstrate robust expression outside of the retina. *gabra6a* is expressed prominently in the photoreceptor cell layer.

## Discussion

In this study, we showed that the GABA_A_ receptor gene family exhibits a number and diversity very similar to those found in mammals. We identified sequences for 23 GABA_A_ receptor encoding genes, and phylogenetic analysis indicates that most isoforms are conserved across vertebrates. We determined the expression of the eight zebrafish α subunit encoding genes, the largest and most diverse gene family, and observed that they are expressed in distinct, often overlapping expression patterns. Taken together, these data argue that larval zebrafish can serve as a useful model to investigate the functional roles of GABA_A_ receptor subtypes within developing neural circuits.

A previous study identified 23 GABA_A_ receptor genes in zebrafish [26]. Our data largely confirm their results, in addition, we identified the previously unreported subunit ρ3b, which is encoded by *gabrr3b*. An α3 and an α3-like gene were also described, but our analysis indicates that zebrafish likely contain only one α3 gene. Given the amount of zebrafish genome data that is available, it seems unlikely that additional GABA_A_ receptor subunit encoding genes exist. However, similar to mammalian systems, sequence data indicate that several zebrafish GABA_A_ receptor encoding genes are alternatively spliced, which further enhances the already extensive diversity of receptor isoforms.

The evolution of the GABA_A_ receptor gene family has been examined by using sequence data from a wide variety of species, including humans, rodents, canary, chicken, frogs, pufferfish, tunicates, *C*. *elegans* and *Drosophila* [43, 44]. Our results are in line with these studies, finding that mammalian θ and ε subunits are unusual when compared to other subunits. Similar to other non-mammalian vertebrates, zebrafish do not have clear orthologs for the θ and ε subunits; their existence outside of the mammalian lineage is unclear [45]. Even among mammalian species, for example comparing humans to rodents, these two subunits are very diverse, which suggests they have evolved at a much faster rate than other subunits [46]. The roles of θ and ε subunits may be unique to mammals or, alternatively, fulfilled in zebrafish and other species using other subtypes. Experiments to investigate the functional ability of zebrafish subtypes to substitute for θ and ε in rodent systems could distinguish between these possibilities.

The α subunits showed a wide range of expression patterns across early zebrafish development (Table 1). On one end of the spectrum are *gabra2a* and *gabra2b*, which appeared to be expressed very broadly at each of the three time-points examined. At the other end of the spectrum is *gabra6a*, which we detected only at 96 hpf and mostly in the photoreceptor cell layer of the retina. The other subunits fall between these extremes, with expression in discrete cells in various brain and spinal cord regions. When GABA_A_ receptor α subtypes are expressed in the same region, they often occupy different domains. For example, *gabra1*, *gabra3*, *gabra4*, and *gabra5* are all expressed in the medulla, but are organized in medial to lateral stripes. The zebrafish medulla has a structural and functional organization in which neurons of shared neurotransmitter, phenotype, age, morphology and functional properties are arranged into stripes [47, 48]. Another example of different GABA_A_ α subtypes occupying different domains within the same region is found in the spinal cord. *gabra4* transcripts were found in ventral cells, likely KA neurons, *gabra5*-expressing cells were observed more dorsally, likely CoPA neurons, and *gabra3* transcripts were detected more broadly in both ventral and more dorsal domains. It is not yet clear how the expression of different GABA_A_ subtypes correspond to the organizational stripes of the hindbrain or cell types in the spinal cord, but it seems likely that some subtypes demonstrate cell-type specific expression and confer distinct responses to GABA. Conclusive cell-type identification will require co-labeling experiments that examine GABA_A_ subunit expression along with specific markers, techniques that determine neuronal identity by revealing cell morphology, and/or precise registration in one of the zebrafish digital brain atlases [49–52].

**Table 1:** Expression of GABA_A_ receptor α subunits at 24, 48, and 96 hours post fertilization.

		α1	α2a	α2b	α3	α4	α5	α6a	α6b
**24 hpf**	dorsal rostral cluster		**•**	**•**					
	hindbrain cluster	**•**	**•**	**•**	**•**	**•**	**•**		
	olfactory placode	**•**							
	spinal cord				**•**	**•**	**•**		
	tegmentum				**•**				
	ventral caudal cluster	**•**	**•**	**•**	**•**	**•**			
	ventral rostral cluster	**•**	**•**	**•**					
**48 hpf**	cerebellum		**•**	**•**					
	diencephalon	**•**							
	hypothalamus						**•**		
	Mauthner cell						**•**		
	medulla oblongata	**•**			**•**	**•**			
	olfactory bulb	**•**							**•**
	pallium				**•**	**•**	**•**		
	subpallium				**•**				
	tegmentum				**•**				
	thalamus				**•**				
**96 hpf**	cerebellum	**•**	**•**	**•**		**•**	**•**	**•**	**•**
	hypothalamus	**•**		**•**			**•**		
	Mauthner cell						**•**		
	medulla oblongata	**•**		**•**	**•**	**•**	**•**		
	olfactory bulb								**•**
	optic nerve	**•**							
	pallium			**•**	**•**	**•**	**•**		
	post-optic commissure			**•**					
	pre-optic region			**•**					
	tectum opticum	**•**				**•**			
	tegmentum	**•**		**•**	**•**				
	thalamus				**•**				
	retina-	**•**		**•**	**•**	**•**		**•**	**•**
	photoreceptors							**•**	

Shaded boxes indicate broad, diffuse expression. Dots indicate punctate expression.

The expression patterns of GABA_A_ α subunits are well-conserved across species. The expression of five α subunits has been described in *Xenopus laevis* during development [53]. Consistent with our observations in zebrafish, α2 was found to be expressed broadly and early in development. In fact, α2 was found to be deposited in eggs as a maternal message. α6 showed the latest onset of expression during development and demonstrated robust photoreceptor expression. Likewise, the expression patterns of α1, α3, and α5 are similar between frogs and zebrafish. These similarities in expression extend to mammalian systems. For example, in rat, similar to zebrafish α2, transcripts were detected relatively early in development and were widespread, and α6 is expressed much later in development and exhibits restricted expression [7]. One notable difference is that in mammals α6 transcripts were detected in the cerebellum and retinal expression has not been reported, whereas in zebrafish α6a exhibits robust retinal expression and α6b is more widespread. It is unclear how much these differences in expression between zebrafish and mammals can be attributed to the various development stages and tissues selected for investigation versus authentic differences in patterns of expression.

Developing zebrafish are a powerful system for neural circuit analysis and, more recently, they have been cultivated as a model of epilepsy [17, 54–58]. Although GABA_A_ receptors are robust regulators of many neural circuits and play a central role in epilepsy syndromes, GABA_A_ receptors have not been well characterized in zebrafish. By reporting the diversity of zebrafish GABA_A_ receptor subunits and describing the expression patterns of the α subfamily during early development, we have laid a foundation to leverage the strengths of the zebrafish system to investigate isoform specific roles of GABA_A_ receptors and further develop zebrafish as a model of epilepsy. In mammalian systems, genetic inactivation of individual α subunits has revealed surprisingly subtle defects compared to what would be predicted from pharmacological blockade. Compensatory upregulation of other subunits is thought to mask some of the effects of inactivating a single subunit [59, 60]. The ability easily mutate multiple genes in zebrafish may help overcome such compensatory changes in expression to shed new light into subtype specific roles of GABA_A_ receptors.

## Supporting information

S1 Fig RT-PCR results suggest that zebrafish contain only one α3 gene. (A) Schematic of *gabra3* cDNA. The portions that are identical to *gabra3*-like and *gabra3* sequences are shaded green. The white region that connects the two was identified through RT-PCR. The location of the three primers used for PCR are shown as numbered arrows. (B) RT-PCR results. Primers 2 and 3 served as a positive control since they amplify sequence from the known *gabra3* region. Primers 1 and 3 amplify a previously unknown region that links *gabra* 3-like and *gabra3* sequence, showing that they are contained within same transcript and likely portions of the same gene.
(PDF)

S1 Table GABA_A_ α subunit antisense RNA probe information. Note- sizes are shown in base pairs. The probe start site is defined here as the start codon of the open reading frame. Negative values indicate that the probe contains 5’ untranslated sequence. Some probes were generated via RT-PCR with a primer that contains an RNA polymerase promoter, so subcloning into vectors wasn’t performed.
(PDF)
