# Supplementary figures and images for: Expression of the eight GABAA receptor α subunits in the developing zebrafish central nervous system
Source: PLoS One. 2018 Apr 27;13(4):e0196083. doi: 10.1371/journal.pone.0196083 (PMC5922542; doi:10.1371/journal.pone.0196083)

A

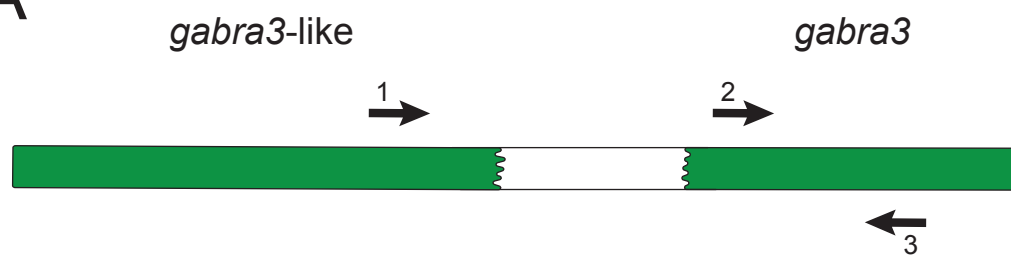

B

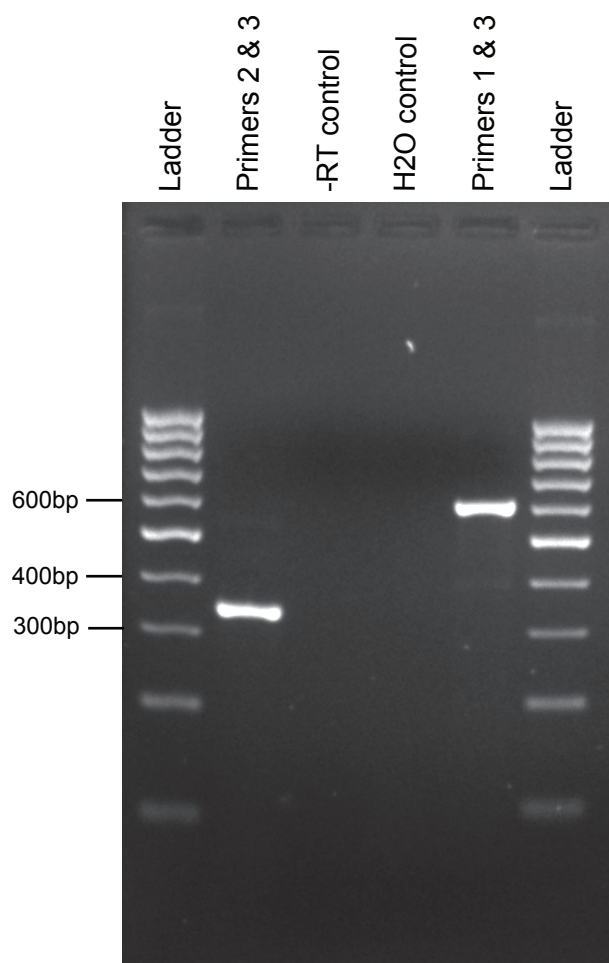

Supplement: S1 Fig — (A) Schematic of gabra3 cDNA. The portions that are identical to gabra3-like and gabra3 sequences are shaded green. The white region that connects the two was identified through RT-PCR. The location of the three primers used for PCR are shown as numbered arrows. (B) RT-PCR results. Primers 2 and 3 served as a positive control since they amplify sequence from the known gabra3 region. Primers 1 and 3 amplify a previously unknown region that links gabra 3-like and gabra3 sequence, showing that they are contained within same transcript and likely portions of the same gene. (PDF) [file pone.0196083.s001.pdf]
