# Supplementary material for: Expression of the eight GABAA receptor α subunits in the developing zebrafish central nervous system
Source: PLoS One. 2018 Apr 27;13(4):e0196083. doi: 10.1371/journal.pone.0196083 (PMC5922542; doi:10.1371/journal.pone.0196083)
Supplement: S1 Table — Note- sizes are shown in base pairs. The probe start site is defined here as the start codon of the open reading frame. Negative values indicate that the probe contains 5’ untranslated sequence. Some probes were generated via RT-PCR with a primer that contains an RNA polymerase promoter, so subcloning into vectors wasn’t performed. (PDF) [file pone.0196083.s002.pdf]

	Accession number	Probe size (bp's)	Probe start	Probe Origin	Vector	Linearization	Transcription enzyme
gabra1	NM_001077326.1	2332	-362	Open Biosystems	pME 18S-FL3	pcr w/ t7/sp6 primers	T7
gabra2a	XM_009307207	294	-324	RT-PCR from zebrafish embryo isolated RNA	PCRII Topo dual	Not1	Sp6
		1183	198	RT-PCR from zebrafish embryo isolated RNA	None	None	T7
gabra2b	XM_017359049	244	-299	RT-PCR from zebrafish embryo isolated RNA	PCRII Topo dual	Not1	Sp6
		521	-263	RT-PCR from zebrafish embryo isolated RNA	none	none	T7
gabra 3	XM_002666071.1	1155	427	Open Biosystems	pBluescript SK (+)	Not1	T7
gabra4	NM_001017822	1674	1	Open Biosystems	pME 18S-FL3	pcr w/ t7/sp6 primers	T7
gabra5	XM_001339475.5	877	-510	Open Biosystems	pME 18S-FL3	pcr w/ t7/sp6 primers	T7
gabra6a	BC059508	2154	-83	Open Biosystems	pBluescript SK (-)	BamH1	T7
		874	1179	Open Biosystems	pBluescript SK (-)	EcoRV	T7
gabra6b	XM_002667357.5	423	249	RT-PCR from zebrafish embryo isolated RNA	pcr4-Topo	Not1	Sp6
		1044	1402	RT-PCR from zebrafish embryo isolated RNA	none	none	T7
